# Enhancing community health system resilience: lessons learnt during the COVID-19 pandemic in Uganda through the qualitative inquiry of the COVID Task Force

**DOI:** 10.3389/fpubh.2023.1214307

**Published:** 2023-11-16

**Authors:** Kiyoko Saito, Makiko Komasawa, Robert Ssekitoleko, Myo Nyein Aung

**Affiliations:** ^1^JICA Ogata Sadako Research Institute for Peace and Development, Tokyo, Japan; ^2^Department of Global Health Research, Juntendo University, Bunkyō, Japan; ^3^College of Health Science, Makerere University, Kampala, Uganda

**Keywords:** health system, community engagement, resilience, COVID Task Force, village health team

## Abstract

**Objective:**

This study aimed to explore the elements of a resilient community health system during the COVID-19 pandemic and discuss whether the frameworks described in previous studies can be applied to real-world situations with those who implemented the Community Engagement Strategy, a strategy to make health systems work in their communities during health crises in Uganda.

**Methods:**

Focus group discussions (22 participants in total) were conducted with COVID Task Force members in four districts in Uganda in March 2022. These districts implemented a Community Engagement Strategy to ensure that health systems in their communities continued to function during health scares, and have been evaluated to ensure that the strategies have been implemented.

**Results:**

A thematic analysis was applied. From the results some factors which can enhance the resiliency of community health systems were identified: including health “knowledge,” “communication,” “governance,” and “resources” health. The most important elements changed depending on the phase of the outbreak. VHTs are the key players in the transition from knowledge-and resource-oriented initiatives to communication and governance by community residents.

**Conclusion:**

COVID-19, a new infectious disease, provides lessons for a resilient community health system. First, the health system should be flexible enough to be able to change the elements on which it is focused, and second, VHTs play an important role in the flexibility of the health system. This suggests that it is time to assess whether VHTs are still able to continue their activities after the pandemic is over, and whether the environment, including financial and non-financial support, has improved.

## 1. Introduction

The COVID-19 pandemic forced African countries, which have limited resources and weak health systems, to reinforce community engagement mechanisms (1, 2). At the outset of the pandemic, the World Health Organization (WHO) prioritized support in for COVID-19 prevention measures in 13 countries in Africa [Algeria, Ghana, South Africa, Tanzania, Kenya, Mauritius, Angola, Cote d’Ivoire, Ethiopia, Democratic Republic of Congo, Nigeria, Zambia, and Uganda (3)] based on their close transport links with China (measured by number of travelers) and urged them to promote community engagement activities (4).

A resilient health system has a high capacity to prepare, manage, and learn from sudden and unpredictable extreme changes affecting the health system, thereby providing quality services, human resources, health financing, up-to-date information, supplies, transportation, communications, and guidance to address a broad range of health challenges (5). For a health system to be truly resilient, both the values and norms of the community and the health system must be consistent. To achieve this, community health workers must be able to use the local population to analyze and guide actions to build and engage community capacity for health development (6).

Corbin et al. stated that the COVID-19 pandemic reconfirmed the need to elevate community engagement, build trust, and support the sustained activity of future health promotion preparedness strategies (7). However, local health departments in many countries have been facing budget and staff cuts, and feel increasingly constrained with regard to the creation of lasting preparedness ties with community partners (8). Bahndari et al. proposed a model for measuring the community resilience of health systems to help communities be better prepared to address health scares (9). However, while many researchers have proposed policies, frameworks, and measurement methods for health-system resilience, few have validated these frameworks and methods.

This study aimed to explore the elements of a resilient community health system during the COVID-19 pandemic and discuss whether the frameworks described in previous studies can be applied to real-world situations with those who have implemented the Community Engagement Strategy, a strategy to make health systems work in communities during health crises. We targeted Uganda, where the WHO prioritized support for COVID-19 prevention measures, and implemented a strategy to elevate community engagement to validate the frameworks in real-world situations.

**Figure 1:**
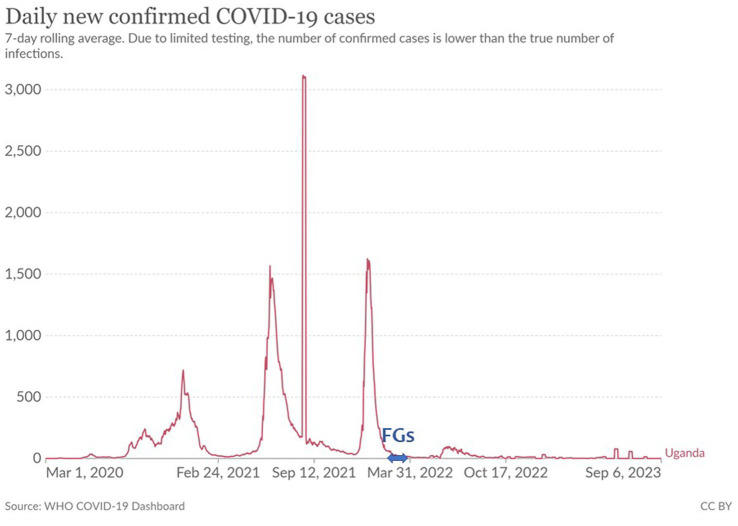
COVID-19 waves and the period of FGs.

In Uganda, a nationwide lockdown was implemented that entailed stopping the normal running of businesses between March 25 and June 30, 2020, and again for 42 days from June 2021, as a response to COVID-19 and concerns about its spread (10, 11). In September 2020, the Task Force for COVID-19 (CTF), presided over by the president of Uganda, established the Community Engagement Strategy (CES) for COVID-19 response. The CTF was a multi-sectoral and multilevel committee established to strengthen the community health system (12–14). CTFs implementing the CES spanned several levels, from the district level down to individual villages. At the village level, the smallest community unit, the Village COVID Task Force (VCTF), was implemented. This involved the participation of full-time paid community health workers (community health teams) (15). To suggest lessons for future resilient health systems, focus groups (FGs) were conducted in March 2022 (Figure 1), more than 1 year after the CES was implemented and the pandemic had subsided to some extent, to analyze reflections in a fresh context.

## 2. Methods

### 2.1. Participants

Four Ugandan regions were selected, where the implementation of the CES was verified by the government (Amuru, Busia, Ngora, and Mukono) (16). Members of the CTFs (22 individuals) in these regions participated in the FGs in the present study (Table 1). Participants were selected from district level members, who supervised the implementation of the CES, and village level members, who implemented it.

**Table 1:** Participants for 4 focus groups.

						District			
						Amuru	Busia	Ngora	Mukono
District level	Status								
	District health coordinator (DHC)					1			1
	Chief administrative officer (CAO)					1	1	1	
	Assistant district health officer (ADHO)					1			1
	Health sub districts surveillance person (HSDS)					1		1	
	Director for health and social services (DHSS)					1		1	1
	Biostatistician (BS)					1	1		
	Medical doctor (MD)						1		
	District health officer (DHO)						1		1
	Resident district commissioner (RDC)							1	
Village level	Village health worker (VHW) (as a member of Village Health Team)					1	1	1	1
				
	Total number of interviews					7	5	5	5

### 2.2. Procedure and analyses

Four FGs were held in March 2022 for 1.5 h and moderated by two researchers experienced in FGDs and qualitative research who had no prior relationship with the participants. After informed consent was obtained, FGDs were conducted in separate groups in each district.

The open-ended question, “The open-ended question, “During the COVID-19 pandemic in your district, what community engagement activities were critical to making the health system work? What were the challenges?” was posed to participants in the local language (Luganda). Participants were also asked to discuss the timeline from the immediate aftermath of the outbreak to the present and voice both the positive and negative aspects of the community health system.

Theme analyses were then performed. Audio recordings of the proceedings were made and notes were taken by a scribe. The audio recordings were then transcribed and coded into meaningful units and summarized for their main themes to be reported through NVIVO (Ver1.7, QSR International). After reviewing and redefining the themes, three members of our research team discussed the subthemes related to each main theme until a complete agreement on the themes was reached and the elements affecting community health system resilience were identified.

## 3. Results

Through these FGs, four categories associated with community engagement during the COVID-19 outbreak were identified: “knowledge,” “communication,” “governance,” and “resources.” Each category was further divided into several subcategories (Table 2). The results also show the extent to which each theme is mentioned in the FGD.

**Table 2:** Categories and % of all mentioned.

		District				
	%	Amuru	Busia	Ngora	Mukono	Total
Knowledge		15.5	19.2	6.4	9.8	24.1
Factual knowledge		6.9	6.4	2.1	0.0	6.7
Training and education		4.0	10.3	4.3	3.3	7.1
Empowerment		4.6	2.6	0.0	6.6	5.2
Communication		32.2	23.1	14.9	19.7	19.8
Open communication		5.7	1.3	2.1	1.6	4.9
Rapid communication		10.9	2.6	6.4	8.2	1
Governance		39.7	28.2	21.3	39.3	15.1
Leadership		1.1	1.3	0.0	1.6	1.5
System		6.3	3.8	6.4	18.0	10.5
Resources		12.1	29.5	53.2	29.5	41.0
Supply		6.3	9.0	17.0	19.7	14.2
Financial		5.2	10.3	21.3	19.7	15.4
Technical		2.9	16.7	27.7	6.6	13.1
Human		4.0	2.6	4.3	3.3	4.9
Other		0.8	0.0	5.4	2.3	1.9

### 3.1. Knowledge

Knowledge was subcategorized into factual, training and education, and empowerment.

#### 3.1.1. Factual knowledge

At the beginning of the pandemic, providing factual knowledge concerning how the virus was spread and how to prevent it from spreading to residents was recognized as important, as COVID-19 was still a new disease at the time. Some residents did not believe that COVID-19 was real. Their beliefs affected the residents’ responses, especially regarding vaccination. Even health workers can have negative attitudes toward vaccination, which discourages communities from receiving vaccination.

Cultural and religious beliefs were mentioned as the greatest impediments facing residents’ uptake of vaccination, known as “the problem of religion,” as follows:

“*… our discussions have helped them understand our reasoning and agree to be vaccinated, but they still experience “religious issues”. Some people say that their religion does not allow vaccination or their culture does not allow it, and I fear that we lack the mechanism to persuade those who do not wish to be vaccinated!*” (CAO, Amuru)

It was also noted that these types of beliefs were further reinforced through discussions in social networking chat groups among individuals with the same beliefs, making it challenging to promote health-related behavioral change.

“*… we experienced issues related to culture in XXX district, everyone in that district uses WhatsApp voice notes to exchange information, and they tend to believe the allegations spread there.*” (BS, Amuru)

#### 3.1.2. Training and education

Among those citing “Knowledge” as an effective approach, “Training and Education” was the most frequently mentioned subcategory (7.1%) during the FGDs. There are two types of “Training and Education” targets, one for residents and one for the village health team (VHT). Many CTFs indicated that during the COVID-19 outbreak, where physical distancing was practiced, VHTs who had close connections with a village’s residents played an important role in maintaining linkages within the community.

“*… in the village, the most reliable people in the village are members of the VHT, and the VHT knows everything about the village, including the total number of residents, their gender, and the ages of the village population.*” (DHC, Mukono)

Therefore, training and education in VHTs have been recognized as key factors contributing to health system resilience.

“*… VHTs are people who actually live in these communities, and they sometimes have to take care of their own families. VHTs therefore don't always think about what should do as VHTs and may need to be re-educated on what they should do as VHTs with regard to community health-related issues. An organization focused on re-educating them should be established.*” (DHO, Mukono)

The VHT members recognize that their role is not specific to health, but extends to support residents’ daily lives.

“*We should help educate community members on how they can better act as a close family.*” (VHW, Amuru)

The responses indicated that the training and education VHT members received to provide effective support to community residents included home-based care management, community management, positive parenting, community (district) surveillance, hygiene promotion at home, management of the 5Ss (staff, staff, space, systems, and social support), surveillance data management, and establishment of a public address system.

#### 3.1.3. Empowerment

The knowledge necessary to empower community residents to enact health-related behaviors involved helping residents distinguish on their own, which patients merited priority access to medical facilities (e.g., pregnant patients) and those for whom home-based care was sufficient.

“*We held radio talk shows and informed the community about the idea of housing pregnant mothers in a facility, and returned them home after birth. Pregnant women should not wait at home to give birth because the road is blocked by a roadblock which prohibits the movement of boda-bodas (private bicycles) and only authorized persons can travel, so even if labor pains occur in the village, they cannot go to the hospital in a timely manner. In this way, we were able to save the lives of most pregnant mothers.*” (RDC, Ngora)

Knowledge of how to handle home-based care is necessary for family members confined to their residences because of the lockdown. Thoroughly trained VHTs initially provided home-based care to residents, encouraging them to care for needy individuals along the way. One VHT member responded as follows:

“*… when you empower communities, communities become able to handle many of their own health issues. In addition, this has proved beyond reasonable doubt that people can support themselves.*” (VHW, Mukono)

### 3.2. Communication

Communication was subcategorized into open communication and rapid communication.

#### 3.2.1. Open communication

CTFs communicated to increase community resilience through open dialog with local community residents and travelers returning from across borders. Since communication with residents primarily involved sharing beliefs, the individuals who took the initiative to open the dialog were an important factor. In most communities, VHTs and religious leaders were recognized as core communicators. Because travelers need to understand the country’s entry/exit controls and restrictions on their behavior, VHTs, security police officers, local community leaders, district officials, and others involved in border entry/exit control all play important roles.

“*… we had a very good relationship with the police, security personnel, local leaders, and district officials. Where enforcement was needed, we encountered no resistance, and the infection was controlled.*” (HSDS, Amuru)

### 3.3. Rapid communication

Regarding rapid communication, including real-time updates on ongoing impact and relief efforts, it was mentioned that communication immediately after the outbreak was difficult, as this was a new virus, and expert testimony was unavailable. CTFs recognized that a delay in rapid communication led to the spread of misunderstandings.

“*It was also necessary for the committee to accept that COVID actually exists and to implement best practice-related SOPs. Digital platforms in particular were a major source of rumors. So much misinformation was being generated using electronic devices that our speed in providing correct information to the community was outstripped by the informal technology network.*” (CAO, Busia)

Based on these experiences, there was a common understanding among community residents that baseless rumors induce the implementation of inappropriate health behaviors, and scientific knowledge should be shared with not only experts but also key players who can accelerate the spread of correct information. Many participants recognized that resilience in such communities, even as it relates to health, is fostered primarily by political leadership and not by health professionals.

“*The political leadership helped a lot in mobilizing the people and fostering the team spirit. I may even go so far as to say that our nontechnical team was a political leader.*” (DHC, Amuru)

### 3.4. Governance

Governance was subcategorized into leadership and system.

#### 3.4.1. Leadership

There have been many references to the importance of leadership demonstrated by certain politicians with stable power at the beginning of the COVID-19 outbreak. However, even in cases where a stable political system was in place, there was substantial confusion initially, so in many instances, politicians trusted by community residents supported the community personally. Furthermore, it was recognized that solid political leadership facilitated the operation of the health system.

#### 3.4.2. System

Participants mentioned the importance of having a health system capable of responding to a broad range of health challenges, including the implementation of a medical delivery system for home-based care, a surveillance system for collecting and reporting patient data from local sites to the central government, a transportation system to ensure patients had free access to medical facilities, a public means of address to communicate with community residents, financial management for appropriate distribution, and a means to control human flow to prevent the spread of the virus into the community. Such systems had been running since before the COVID-19 outbreak, and it was mentioned that helping partners work with their existing systems was a key to ensuring community resilience.

“*When the pandemic was first reported, the government simply scaled up existing systems, and our partners supplemented the system in place to boost output. As such, most tools we used were already in place.*” (BS, Busia)

### 3.5. Resources

Resources were subcategorized into supply, technical, financial, and human resources.

#### 3.5.1. Supply

Among the resources provided, critically necessary supplies (e.g., food, water, and hygiene products, including masks and alcohol-based sanitizers) were most useful immediately after the outbreak. Because these resources can be provided without a health system, they were offered to the community by individual partners, as well as NPOs, NGOs, and other organizations, even in the chaos following the outbreak when the system was not functioning. Individual donors included not only community leaders, but also ordinary people who stored food. Notably, food was the most frequently provided resource, as it is fundamental to life.

“*… in the chaos immediately following the outbreak, what is most important for us is survival, the protection of life, and having something to eat.*” (DHSS, Mukono)

After ensuring direct support from community residents, the focus gradually shifted to providing resources to the health system. These kinds of resources were mainly supplied by the government at the beginning, but eventually ran out, and the main providers shifted to other organizations (e.g., NGOs and NPOs).

Equipment such as thermometers, gloves, and jackets have been mentioned as very useful for facilitating VHT activities and complementing the limitations of hospital resources and home-based care.

A facility called the “Isolation Center,” opened by the village government, worked well to isolate COVID-19 patients and prevent the widespread spread of COVID-19. NGOs and NPOs provide facilities with tents, chairs, and mattresses.

#### 3.5.2. Technical

The resources of particular note in the COVID-19 outbreak were technical resources such as digital tools and automobiles for VHTs. To monitor the infection situation in the community, it was necessary to create a system that would allow VHTs to visit each household and report the situation, including hospital referrals, to the health center at the district level as quickly as possible.

Transportation services were required to allow VHTs to visit each household and reach the district committee. In addition, automobiles delivered patients to hospitals, as few ambulances are available in the community setting.

“*Motorcycles were given to VHTs to hasten their movement.*” (DHO, Ngora)

Most CTFs mentioned that digital tools such as mobile phones and tablets were useful for rapid surveillance, including monitoring at the village level and reporting to the district level. VHTs were able to send data via these digital tools without needing to visit the local government offices.

“*… smartphones have also been helpful; the VHT no longer has to travel from each village to a government facility to report surveillance results.*” (VHW, Amuru)

#### 3.5.3. Financial

Among resources, financial elements were the most frequently mentioned (15.4%). Although government funding was available to facilitate CTFs’ “COVID facilitation fund” activities at the beginning of the outbreak, these funds dried over time, eventually reaching zero in several districts.

In addition to the limited size of the government budget, the inadequacy of the fund utilization system was mentioned. The guidelines for the fund allocation system, called the “Integrated Financial Management System” by the Ministry of Finance, were not properly followed by district and village governments, resulting in different directives at each level, which made it difficult for VHTs to allocate the budget properly.

“*The VHT was not instructed on when to disburse aid funds. And now here we are, with the auditors pointing out that the funds and aid money are not being used, thus causing confusion! For this reason, during the second wave of relief, people felt demoralized.*” (DHSS, Mukono)

However, it was recognized that financial support from NPOs, NGOs, and other partners was utilized properly, with clear objectives and guidance on how to use the funds. These funds were provided for various purposes, such as obtaining supplies and technical resources, but the most effective use was for community mobilization and funding front-line workers such as CTF members.

“*We are very proud of XXX (NGO) for setting a good course for housing and managing COVID patients in the village by strengthening community participation strategies, mobilizing, educating and enlightening CTFs, and implementing a home care management system.*” (RDC, Ngora)

The community recognized that even with limited financial resources, VHTs could work voluntarily and support local residents in areas where they had already existed and functioned well in the community.

“*We had enough health workers, but they were not being adequately utilized. However, when the pandemic hit, people rose up and stood hand in hand with them.*” (VHW, Amuru)

Furthermore, VHTs worked well because they had established collaborative relationships with stakeholders that were meant to be sustainable to build community resilience. According to the FGDs, VHTs worked closely with the local governments of villages and districts, domestic and international NPOs, NGOs, and other organizations related to health, finance, food, digital resources, education, and apparel.

“*These good working relationships that the pandemic has helped establish between the VHTs and healthcare workers and district authorities, as well as other organizations, should continue. We should definitely continue to work together with that good working spirit.*” (VHW, Busia)

## 4. Discussion and conclusion

We explored the perceptions of key players involved in community engagement strategies in Uganda concerning the resilience of community health systems during the COVID-19 outbreak. Knowledge, communication, governance, and resources were identified as the elements of community engagement that contribute to health system resilience.

Bhandari and Alonge (8) insisted that knowledge, financial resources, human, social, and physical capital were relevant in all types of shock, including community-level health scares, and our results are consistent with those previous findings. Furthermore, our results revealed details concerning the contribution of each element. Resources and knowledge were recognized as the elements that most strongly contributed to a resilient health system. During the outbreak, a key feature of this pandemic was the lockdown, and the presence of resources was recognized as critical when logistics and human flow were disrupted, and resource constraints became more severe. While many studies emphasized the importance of dialog, joint problem-solving, and action by many stakeholders, dialog can only be achieved if the environment necessary to sustain the lives of community members is first secured. Some studies have insisted that government initiatives are not representative of true community engagement (15); however, our results showed that the government lockdown restricted mobility, so only the government could ensure the necessary logistics, and our findings suggest that some issues require government initiatives.

In times of turmoil, it is necessary to provide the community engagement system time to reboot or strengthen, and we believe that the leading districts implementing the community engagement strategy in Uganda played a role in this process. Initially, they worked with individuals and organizations who provided supplies such as food, water, and other resources for residents’ survival, distributed them to community residents, and then shifted their focus to helping residents to take an active role in their own health.

Communication and governance are mentioned less frequently than are knowledge and resources. This may be because communication and governance were mentioned less often when CTF members discussed the immediate aftermath of the outbreak and more often when they discussed the later aftermath. This result indicates that the most important elements can change depending on the phase of the outbreak. The key players in the transition from knowledge-and resource-oriented initiatives to communication and governance by community residents were the VHTs (as members of the CTFs), which included members of the local community. The VHTs were recognized as having a good grasp of the community situation, down to the smallest detail, such as the circumstances of individual households. They were able to consult on decisions regarding the allocation of resources and budget for community issues. In addition, these teams were also involved in responding to previous outbreaks, such as Ebola, SARS, and malaria. VHTs are well aware of various stakeholders’ networks and are deeply involved in the existing health system. Therefore, VHTs played an essential role in fostering community engagement to ensure a resilient health system during the outbreak.

The frequent mention of certain resources needed for VHT, such as training and education, digital tools, and automobiles, underscored the role of VHTs as community hubs. Furthermore, we found that for VHTs to function as hubs, the smooth running of activities required a healthy system capable of handling a number of day-to-day issues supporting the local population, not only health-wise but also with regard to finances and supplies.

VHTs handle various health-related issues, such as data collection and reporting, surveillance, referral to hospitals, home-based care for COVID-19, and other matters such as treatment for HIV infection and malaria, pregnancy management, and maternal and child healthcare.

Although many studies have identified communication as an important aspect, we revealed that rapid communication is particularly important. To provide up-to-date information, digital tools are used to collect information from the government and spread that information through media such as radio talk shows. However, the speed of information diffusion was clearly faster in informal networks, such as among residents and religious communities, than in communications from direct government sources. This disparity in speed led to the spread of misconceptions and rumors, resulting in an aversion to vaccination. Many African countries have used digital tools for the COVID response. Rwanda and South Africa leveraged a digital tool to complement traditional contact tracing methods, accommodate an increased workload, and maintain efficiency, and insisted on the need for a robust digital platform to host and share data across jurisdictions (12).

The Ugandan results demonstrated the importance of organically harmonizing new digital and existing analog strategies, which will be a key to the resilience of future health systems. Going forward, CTFs can play the role of harmonizer, as they recognize the need for rapid collection and integration of health-related data in rural areas, and the challenges of achieving a digitalized health system.

Inadequate numbers of qualified healthcare workers, logistics, health information surveillance, governance, and drug supply systems were recognized as weak points during the Ebola virus outbreak (16). The present results suggest that during the COVID-19 outbreak, most of these elements were recognized as important and received particular focus from CTFs. This implies that CTFs were trying to apply the lessons learned during the Ebola outbreak, and the community engagement strategy with CTFs at its core, who were well aware of Uganda’s weaknesses in infectious disease control, worked effectively in Uganda.

In summary, the results indicate that the role of VHTs is critical for a resilient community health system. This suggests that now that the pandemic has been contained, it is time to reassess whether VHTs can continue fulfilling their role.

Uganda can learn from good cases in other countries, such as Tanzania, where a combination of financial and non-financial incentives was shown to be effective for boosting the motivation and satisfaction of health workers (17).

## 5. Limitations

As the participants of this study were CTF members in districts implementing the Uganda CES, the results are significant in terms of involving real voices from practitioners. However, the sample size was limited, and focus group interviews were significant for stimulating discussion, but negative in that participants’ responses influenced other participants. Therefore, a questionnaire survey based on our results with a resilient community health system indicator was conducted to verify the robustness of our findings.

## Data availability statement

The raw data supporting the conclusions of this article will be made available by the authors, without undue reservation.

## Ethics statement

Written informed consent was obtained from the individual(s) for the publication of any potentially identifiable images or data included in this article.

## Author contributions

Material was prepared using KS and MK. Collection was performed using RS and KS. The first draft of the manuscript was written by KS. MA and MK critically contributed to the revision of the content. All authors contributed to the article and approved the submitted version.
